# Immune-Complexed Adenovirus Induce AIM2-Mediated Pyroptosis in Human Dendritic Cells

**DOI:** 10.1371/journal.ppat.1005871

**Published:** 2016-09-16

**Authors:** Karsten Eichholz, Thierry Bru, Thi Thu Phuong Tran, Paulo Fernandes, Hugh Welles, Franck J. D. Mennechet, Nicolas Manel, Paula Alves, Matthieu Perreau, Eric J. Kremer

**Affiliations:** 1 Institut de Génétique Moléculaire de Montpellier, CNRS 5535, Montpellier, France; 2 Université de Montpellier, Montpellier, France; 3 iBET- Instituto de Biologia Experimental e Tecnológica, Oeiras, Portugal; 4 Instituto de Tecnologia Química e Biológica, Universidade Nova de Lisboa, Oeiras, Portugal; 5 Division of Immunology and Allergy, University of Lausanne, Lausanne, Switzerland; 6 Institut Curie, INSERM U932, Paris, France; La Jolla Institute for Allergy and Immunology, UNITED STATES

## Abstract

Human adenoviruses (HAdVs) are nonenveloped proteinaceous particles containing a linear double-stranded DNA genome. HAdVs cause a spectrum of pathologies in all populations regardless of health standards. Following repeat exposure to multiple HAdV types, we develop robust and long-lived humoral and cellular immune responses that provide life-long protection from de novo infections and persistent HAdV. How HAdVs, anti-HAdV antibodies and antigen presenting cells (APCs) interact to influence infection is still incompletely understood. In our study, we used physical, pharmacological, biochemical, fluorescence and electron microscopy, molecular and cell biology approaches to dissect the impact of immune-complexed HAdV (IC-HAdV) on human monocyte-derived dendritic cells (MoDCs). We show that IC-HAdV generate stabilized complexes of ~200 nm that are efficiently internalized by, and aggregate in, MoDCs. By comparing IC-HAdV, IC-empty capsid, IC-Ad2ts1 (a HAdV-C2 impaired in endosomal escape due to a mutation that impacts protease encapsidation) and IC-AdL40Q (a HAdV-C5 impaired in endosomal escape due to a mutation in protein VI), we demonstrate that protein VI-dependent endosomal escape is required for the HAdV genome to engage the DNA pattern recognition receptor AIM2 (absent in melanoma 2). AIM2 engagement induces pyroptotic MoDC death via ASC (apoptosis-associated speck protein containing a caspase activation/recruitment domain) aggregation, inflammasome formation, caspase 1 activation, and IL-1β and gasdermin D (GSDMD) cleavage. Our study provides mechanistic insight into how humoral immunity initiates an innate immune response to HAdV-C5 in human professional APCs.

## Author Summary

While numerous studies have addressed the response to primary virus infections, we know relatively little about the interplay between recurrent and/or persistent infections and the memory humoral immune response on professional antigen-presenting cells. Immune complexed-adenoviruses are present in patients suffering from adenoviremia. In addition to the impact of HAdV infections on healthy and immune suppressed hosts, humoral immunity hampers the use of human adenovirus vectors during gene transfer. Our study shows that anti-adenovirus humoral immunity engages an innate immune response to cause pyroptosis of antigen-presenting cells. The downstream effects of this cells death is unknown and may impact the stimulation and differentiation of T cells into an inflammatory phenotype that may be associated with the complications during adenovirus disease and adenovirus vector use. Our study generates insight into how humoral immunity shapes the response to adenoviruses in healthy and immune-compromised individuals, during human adenovirus-based vaccine use, and during antibody therapy.

## Introduction

Adenoviruses (AdVs) have a 28–42 kilobase pair double-stranded DNA genome encapsidated in a nonenveloped proteinaceous icosahedral shell. In immune-competent individuals, human AdVs (HAdVs) (of which there are approximately 70 types) cause self-limiting respiratory, ocular and gastro-intestinal tract infections. After repeated encounters, we typically develop multifaceted long-lived memory immune responses [1–3] that efficiently blunt HAdV-induced disease. In spite of the robust cross-reacting cellular and humoral immune responses, HAdVs can establish subclinical persistent infections that last for years, if not decades [4,5]. Not surprisingly, HAdV type-specific humoral immunity before hematopoietic stem cell transplantation is predictive of escape of the same type during immune suppression [6].

Given the ubiquitous humoral immunity against HAdV, it is not surprising that immune-complexed (IC) HAdVs (IC-HAdVs) are detected in some patients with HAdV infections [7–9]. IC-viruses can form during prolonged viremia, secondary infections, in primary infection when a cross-reactive humoral response exists, and antibody (Ab)-based antiviral immunotherapies. In B-cell competent/T-cell compromised patients, the loss of control of persistent HAdV infection might trigger, or exacerbate, graft-versus host disease [10–12]. While IC-antigens are efficient stimulators of dendritic cell (DC) maturation [13], most studies have used prototype antigens that have little impact on processing of the immune complex. How IC-HAdVs are processed and affect DC function are unknown.

DCs are equipped with a broad set of pattern recognition receptors (PRR) to detect pathogen-associated molecular patterns (PAMP) at the plasma membrane, in vesicles, or in the cytosol. Foreign DNA is a PAMP that can be detected in endolysosomes by Toll-like receptor 9 (TLR9) and in the cytosol by absent in melanoma 2 (AIM2) [14]. Nucleic acid sensing by TLRs stimulates a broad set of signaling pathways, notably, the NF-κB, AP-1, interferon-regulating factors pathway and/or inflammasome formation [15]. The inflammasome is a multiprotein platform formed in the cytosol consisting of a PRR, ASC (apoptosis-associated speck protein containing a caspase activation/recruitment domain), and caspases [16]. Upon induction, inflammasome sensors AIM2 or nucleotide-binding domain and leucine-rich repeat containing protein 3 (NLRP3) initiate aggregation of ASC that in turn prompts recruitment [17] and proximity-induced caspase 1 auto-activation [18], and pro-1β and gasdermin D (GSDMD) cleavage [19]. Stimuli that induce inflammasome activation can be as diverse as cytosolic DNA [18], extracellular adenosine triphosphate [20], plasma membrane rupture [21], and/or lysosomal rupture releasing cathepsin B into the cytosol [22].

Inflammasome-mediated effects play contrasting roles in vaccination [23,24], during infection in immune-compromised hosts [25], and auto-inflammatory disease [26]. Of note, inflammasome activation can result in pyroptosis [27], an inflammatory form of cell death characterized by caspase 1 activation and rapid loss of plasma membrane integrity due to GSDMD cleavage [19]. In PAM3CSK4-primed THP-1 cells, super-infection with HAdV-C5 causes endosomal lysis and cathepsin B release, which is accompanied with mitochondrial stress, ROS formation, NLRP3 inflammasomes, and IL-1β maturation and secretion [28–30]. Interestingly, IL-1β secretion is higher in primed THP-1 cells when challenged with HAdV-C5 pre-incubated with human serum [30,31], but the underlying mechanism is unknown. In addition, for three decades vectors derived from human and nonhuman AdV types have been developed as candidates for infectious disease vaccination, and pre-clinical results have often been impressive. However, use in humans has not been without concerns [32,33]: two proof-of-concept HIV vaccine trials (STEP and Phambili) using a HAdV-C5-vectored vaccine, were interrupted due to lack of efficacy and, unexpectedly, an increased risk of HIV acquisition in some vaccinees [34,35]. Importantly, early HIV acquisition in vaccinees correlates with pre-existing B and T-cell immunity targeting HAdV-C5 [35]. Potential explanations for higher HIV infection rates are that IC-HAdV are potent driver of DC maturation, which in turn induces CD4 T-cell activation and proliferation [36], or proliferation of anti-AdV Th17 CD4 T cells with mucosal homing [37–39], which makes them primed targets for HIV infection.

The ubiquitous HAdV humoral immunity and its impact on omnipresent wild type HAdV encounters and during HAdV vector use, led us to investigate the molecular and cellular events following IC-HAdV uptake by human professional antigen-presenting cells (APCs). To address this, we characterized IC-HAdV composition, internalization, trafficking and processing, the role of protein VI endosomal lysis, and the PRR involved in HAdV-C5 detection. We show that neutralizing Abs (NAbs) cluster multiple HAdV-C5 particles and render capsid more stable at acidic pH. In monocyte-derived DCs (MoDCs), IC-HAdV stimulate vesicular DNA sensor TLR9 inducing TNF production, and then escape into the cytosol in a protein VI-dependent manner. There, the HAdV-C5 genomes activate the AIM2 inflammasome formation, which leads to caspase 1 activation, IL-1β processing, GSDMD cleavage, and loss of cell membrane integrity. Our study provides a molecular basis to understand the source of the adverse effects that have been observed in multiple clinical scenarios.

## Materials and Methods

### Cells and culture conditions

Human blood samples were obtained from anonymous donors at the regional blood bank (EFS, Montpellier, France). An internal review board approved the use of human blood samples. MoDCs were generated from freshly isolated CD14^+^ monocytes in the presence of 50 ng/ml granulocyte-macrophage colony-stimulating factor (GM-CSF) and of 20 ng/ml interleukin-4 (IL-4) (PeproTech, Neuilly sur Seine, France) [40]. MoDC stimulations were performed at 6 d postisolation of monocytes. THP-1-ASC-GFP cells [41] were cultured like MoDC. 911 cells [42] and 293T cells [43] were grown in Dulbecco’s modified Eagle’s medium (DMEM) supplemented with 10% fetal bovine serum (FBS).

### Adenovirus vectors

Adβgal is a ΔE1/E3 HAdV-C5 vector harboring a *lacZ* expression cassette [44]. AdL40Q is a HAdV-C5-based vector with a leucine to glutamine mutation of amino acid 40 in protein VI that decreases its membrane lytic activity [45]. AdL40Q was used within 3 months post-propagation/purification [46]. Ad2ts1, from the closely related HAdV-C2 type, harbors a mutation in the protease which prevents encapsidation and this results in several unprocessed capsid proteins (IIIa, VI, VII, VIII, TP and mu) and a hyperstable capsid [47,48]. Alexa555- and Alexa488-HAdV-C5 (referred to as HAdV-555 and HAdV-488, respectively) were generated from Adβgal by using a Alexa555 or Alexa488 Protein Labeling Kit (Life Technologies) as previously described [49]. Vectors were purified to homogeneity by two CsCl density gradients as previously described [44]. Empty HAdV-C5 capsid are generated during vector production. They are less dense and are easily separated from intact capsids by isopycnic CsCl centrifugation.

### Immune complex formation and DC stimulation

MoDCs (4 x 10^5^ in 400 μl of complete medium) were incubated with HAdV-C5 vector, or IC-AdV (both 2 x 10^4^ physical particles (pp)/cell, unless indicated). We generated IC-HAdVs by mixing the virus (8 x 10^9^ pp, or 2 x 10^4^ pp/cell) with 2.5 μl of IVIg (human IgG pooled from between 1,000 and 50,000 donors/batch) (Baxter SAS, Guyancourt, France) for 15 min at room temperature. IVIg is used in patients with primary or acquired immune deficiencies as well as autoimmune diseases. A synthetic oligodeoxynucleotide (ODN) containing the immunosuppressive motif TTAGGG that block TLR9 and AIM2 signaling (ODN A151 [50]) was added 2 h before stimulation. ODN A151 was synthesized by IDT. TLR4 agonist lipopolysaccharide (LPS) (Sigma) and NLRP3 inducer nigericin (Invivogen) was used at 100 ng/ml and 10 μM, respectively. Z-VAD (InvivoGen), WEHD (Santa Cruz) and YVAD (InvivoGen) were added 1 h before stimulation at 20 and 100 μM. MoDCs (4 x 10^5^ in 400 μl of complete medium) were incubated with HAdV-C5 vector, or IC-HAdV (both 2 x 10^4^ physical particles (pp)/cell, unless indicated) for indicated time.

### Quantification of virus internalization

Virus internalization was determined by qPCR as previously described [51,52]. Briefly, MoDCs were incubated on ice with Adβgal, IC-HAdV or IgG for 30 min prior to incubation for up to 6 h at 37°C. To distinguish between extracellular and internalized virus, samples were divided into two equal aliquots. In one of these, cell surface-associated virus was removed by acid wash in one sample as previously described [52]. Briefly, cells were washed three times with ice-cold PBS followed by 0.2 N acetic acid, 0.5 M NaCl, pH 2.5. Residual plasma membrane-associated virus was removed by incubation with 2.5 mg/ml pronase XIV with 0.025 μg/ml DNase in RPMI at 4°C for 1 h. DNA from acid-washed and the mock-treated samples were extracted as previously described [53]. Viral genomes/cell were compared to standard curves and then normalized to GAPDH.

### RNA interference

Lentivirus vectors harboring shRNA expression cassettes were purchased from Open BioSystems or designed in house. Targeted genes and shRNA sequence clones are listed in S1 Table. To prepare lentivirus vectors, nearly confluent 293T cells were cotransfected with pLKO-shRNA, pCMV-ΔR8.91 (gag/pol) and pCMV-VSVG using TurboFect (Thermo Scientific). Helper particles were produced in 293T co-transfected with pSIV3+ and pCMV-VSVG [54]. One day posttransfection medium was replaced by fresh medium. Supernatants were collected 36 h posttransfection and used immediately to transduce freshly isolated monocytes plated at 2 x 10^6^ cells/well of a 6-well plate in 2 ml of complete medium. One milliliter of 0.45 μm-filtered lentivirus vector supernatant made with 2 shRNAs clones targeting the same gene and 0.5 ml of supernatant containing helper particles were added to each well. Polybrene (1 μg/ml), IL-4 (20 ng/ml) and GM-CSF (50 ng/ml) were immediately added. Approximately 80% of DCs were transduced (as observed by flow cytometry) 6 d postincubation as observed by flow cytometry when using lentivirus vectors harboring a GFP expression cassette. shRNA knockdown efficacy was checked by western blot. THP-1-ASC-GFP cells were also transduced by lentiviral particles coding for shRNAs with 1 μg/ml polybrene. Transduced THP-1-ASC-GFP cells were kept undifferentiated until their use in pyroptosis assays.

### Pyroptosis assay

THP-1-ASC-GFP cells were differentiated into DCs over 6 d in complete medium supplemented with IL-4 (20 ng/ml) and GM-CSF (50 ng/ml) (PeproTech). Differentiated THP-1-ASC-GFP cells were plated in 24-well plates at 10^5^ cells/well in 200 μl of complete medium. These cells were pretreated with Z-VAD-FMK (50 μM) for 30 min to prevent cell death, and then exposed to HAdV-C5 vector, IVIg or IC-HAdV for 1 h. Whole cells were centrifuged onto glass slides at 650 RPM for 5 min using cytospin, washed once with PBS, fixed with 4% paraformaldehyde (15 min at room temperature), mounted with DAKO fluorescent mounting medium containing 10 μg/ml DAPI (Sigma) and observed by fluorescent microscopy [41]. GFP loss (decrease in mean fluorescent index) and extracellular LDH activity were used as a proxy for cell loss of membrane integrity because of the ease of measuring GFP fluorescent intensity by flow cytometry and LDH activity in the supernatant.

For quantification of pyroptosome formation in MoDC, MoDC were seeded at 1.2 x 10^5^ cells/well in 500 μl complete medium on poly-L-lysine (Sigma)-coated coverslip in a 24-well plate. The cells were attached to the coverslip by centrifugation at 1000 RPM and incubated for several hours at 37°C/5% CO_2_. MoDC were incubated with 20 μM Z-VAD or 100 μM ODN A151 where indicated for 2 h and then challenged by DNA transfection or 20,000 pp/cell IC-HAdV, HAdV-C5 or 1.5 μl of IVIg for 3 h. Cells were fixed with 4% paraformaldehyde, permeabilized with 0.1% Triton X-100 and stained with mouse anti-ASC antibody (dilution 1:100) and corresponding fluorescently-labelled secondary antibody. Cover slips were mounted with DAKO fluorescent mounting medium containing 10 μg/ml DAPI. For quantification, 70 to 700 MoDC from 2 donors were manually counted using confocal microscopy per condition to identify puncta or diffuse ASC signals. Ratios between cells and cells containing pyroptosomes were calculated.

### Western blot and antibodies

Total protein extracts were prepared by using RIPA buffer (10 mM Tris-Cl (pH 8.0), 1 mM EDTA, 0.5 mM EGTA, 1% Triton X-100, 0.1% sodium deoxycholate, 0.1% SDS, 140 mM NaCl) plus protease inhibitors (Roche) and phosphatase inhibitor (Roche). Protein lysates were used for SDS PAGE followed by western blot analyses. Primary antibodies were detected using HRP-conjugated secondary antibodies against rabbit or mouse (Sigma) at a dilution of 1:10000. Antibodies used in this study for western blot include: mouse anti-β-tubulin (T4026, Sigma), (dilution 1:15000), rabbit anti-MyD88 (Ab2064, AbCam) (dilution 1:500), rabbit anti-AIM2 (Ab76423, AbCam) (dilution 1:1000), rabbit anti-TLR9 (Ab52967, AbCam) (dilution 1:250), mouse anti-AP3B1 (WH0008546M6, Sigma) (dilution 1:500), rabbit anti-IL-1β (#sc-7884, Santa Cruz Biotechnology) (dilution 1:500), anti-GSDMD (G7422, Sigma) (1:1000).

### Quantification of mRNA

Expression levels of NLRP3 and AIM2 genes were analyzed using qRT-PCR. Total RNA was isolated from DCs using the High Pure RNA isolation Kit (Roche) with a DNase I treatment during the purification and eluted in 50 μl of DEPC-treated water. Reverse transcription was performed with the Superscript First-Strand Synthesis System (Invitrogen) using 8 μl of total RNA and random hexamers. The cDNA samples were diluted 1:6 in water and analyzed in triplicate using a LightCycler 480 detection system (Roche). SYBR green PCR conditions were 95°C for 5 min and 45 cycles of 95°C for 15 s, 65°C or 70°C for 15 s, and 72°C for 15 s using GAPDH sequences as standard. Primer sequences were as follows for NLRP3 (5’-CCTCTCTGATGAGGCCCAAG-3’ (NLRP3 forward) and 5’-GCAGCAAACTGGAAAGGAAG-3’ (NLRP3 reverse)) at 65°C, AIM2 (5’-GCTGCACCAAAAGTCTCTCC-3’ (AIM2 forward) and 5’-TCAAACGTGAAGGGCTTCTT-3’ (AIM2 reverse)) at 65°C, IL-1β (5’-AAACAGATGAAGTGCTCCTTCC-3’ (IL-1β forward) and 5’-AAGATGAAGGGAAAGAAGGTGC-3’ (IL-1β reverse) at 65°C, GAPDH (5’-ACAGTCCATGCCATCACTGCC-3’ (GAPDH forward) and 5’-GCCTGCTTCACCACCTTCTTG-3’ (GAPDH reverse) at 70°C. Relative gene expression levels of each respective gene were calculated using the threshold cycle (2^-ΔΔCT^) method and normalized to GAPDH [55].

### IC-HAdV size

The size of HAdV-C5 and IC-HAdV complexes was determined using a light scattering device (NanoSight NS500, NanoSight, Amesbury, UK). After the incubation of HAdV-C5 with NAb, samples were diluted with Dulbecco’s PBS to reach a concentration of approximately 1 x 10^7^ particles/ml). NanoSight LM10 recorded 60 s sample videos, which were then analyzed in the Nanoparticle Tracking Analysis (NTA) 2.0 Analytical software release version build 0125 (NanoSight). The NTA software technology uses the properties of both light scattering and Brownian motion to obtain the size distribution and concentration measurement of particles in liquid suspension [56]. All samples were run twice and analyzed separately.

### Flow cytometry

Surface levels of CD40 and CD86 were assessed by flow cytometry as previously described [40]. Cell membrane integrity was assessed by collecting cells by centrifugation 800 x g, the cell pellets were resuspended in PBS, 10% FBS, propidium iodide (Sigma) or 7-aminoactinomycin D (Becton-Dickinson) and analyzed on a FacsCalibur flow cytometer (Becton-Dickinson).

IC-HAdV mean diameter was assessed using an LSR Fortessa flow cytometer (Becton-Dickinson) for microparticle analysis as previously described [57]. HAdV-555 alone, IgG or IC-HAdV-555 were identified by side scatter and fluorescence. Size was determined with TransFluoSpheres 1.0 μm (Life Technology) and SpheroCalibration Particles 3 μm (Becton-Dickinson).

### Caspase 1 assay

Caspase 1 activation was determined using the FLICA Apoptosis Detection Kit for Caspase-1 (Immunochemistry Technologies) according to the manufacturer’s guidelines with slight modifications. In brief, MoDC were preincubated with 2.5 μM FAM-YVAD-FMK FLICA for 30 min and incubated to 20,000 pp/cell HAdV-C5, IVIg, IC-HAdV, LPS or LPS/nigericin as described in the section “Immune complex formation and DC stimulations” for 3 h. Subsequently, cells were collected by centrifugation and washed twice with the apoptosis buffer. Fluorescence of the cells was assessed using flow cytometry.

### LDH assay

LDH release was determined using the LDH cytotoxicity kit (Pierce) according to the manufacturer’s guidelines. Briefly, 50,000 MoDC were plated in 100 μl in 96-well plates and exposed to 20,000 pp/cell HAdV-C5, IVIg, IC-HAdV or LPS/nigericin in triplicates as described in the section “Immune complex formation and DC stimulations” for 6 h. LDH activity in cell culture supernatants was detected using the kit reagents and calculated according to the 100% LDH release control from lysed cells.

### Electron and fluorescence microscopy

MoDCs were incubated for 3 h with HAdV-C5, IVIg or IC-HAdV and subsequently prepared for thin section transmission electron microscopy (TEM) to visualize internalized immune-virus complexes as previously described [58]. For confocal immunofluorescence microscopy, MoDCs were seeded on poly-L-lysine coated cover slips for 60 min at 37°C followed by incubation with HAdV-C5 or IC-HAdVs. Postincubation, cells were fixed, permeabilized and stained with indicated primary antibodies and fluorophore labeled secondary antibodies. Confocal microscopy analysis was carried out using a Leica SP5 (Leica). Primary antibodies for immunofluorescence included rabbit anti-AIM2 (HPA031365, Sigma) (dilution 1:100); mouse anti-ASC (D086-3, MBL) (dilution 1:100) mouse anti-p62/sqstm1 (Ab56416, AbCam) (dilution 1:100) mouse anti-galectin-3 (B2C10, Becton-Dickinson Pharmingen) (dilution 1:100). Blue, green, red, and far-red fluorescence were acquired sequentially.

### Cytokines secretion

Supernatants were collected and secretion of TNF and IL-1β was quantified by ELISA using OptEIA human TNF ELISA Kit (Becton Dickinson) and human IL-1β/IL-1F2 DuoSet ELISA (R&D systems) following the manufacturer’s instructions.

### Serum depletion

Antibodies against HAdV-C5 capsid proteins were depleted from IVIg using Pure proteome NHS FlexiBind Magnetic Beads (Millipore). One hundred microliters of 20% beads were incubated overnight with 30 μg of recombinant capsid proteins on a rotary shaker at 4°C. Beads were collected with a magnetic rack (Millipore) and washed 7 times with 1 ml PBS to remove unbound proteins. After a final wash, the beads were resuspended in 200 μl of NAbs diluted 1:4 in PBS and incubated on a rotary shaker overnight at 4°C. Beads were then removed from depleted serum with a magnetic rack. Antibodies bound on protein-bead complexes were eluted by incubation of beads in 165 μl of 0.1 M glycine (pH 2.7) for 2 min at room temperature and beads were removed from suspension with a magnetic rack. Thirty-five microliters of 1 M Tris-Cl, pH 8 was added to neutralize the pH of the solution. Mock controls were the same beads incubated in PBS instead of HAdV-C5 capsid proteins.

### Capsid stability assay

HAdV-C5 vector alone (6 x 10^9^ pp), IVIg (10 μg) or IC-HAdV were exposed to pH 5 (sodium acetate), pH 6 (MES), pH 7 (HEPES) or pH 9 (carbonate buffer) buffers (15 μl final volume) and capsid disassembly was assessed by DNA detection using Picogreen fluorescent dye (Invitrogen). The ABI prism 7900HT PCR machine (Applied Biosystems) was programmed to measure fluorescence (λex = 488 nm and λem = 520 nm) every 2.5°C from 30 to 70°C.

### Statistical analysis

All experiments were performed a minimum of three independent times and expressed as mean values ± SD unless otherwise stated. Comparisons of groups for statistical difference were performed using Student’s t-test. A *p* value of <0.05 is denoted as significant.

## Results

### Anti-hexon antibodies crosslink and stabilize HAdV-C5, and induce DC maturation

**Fig 1:**
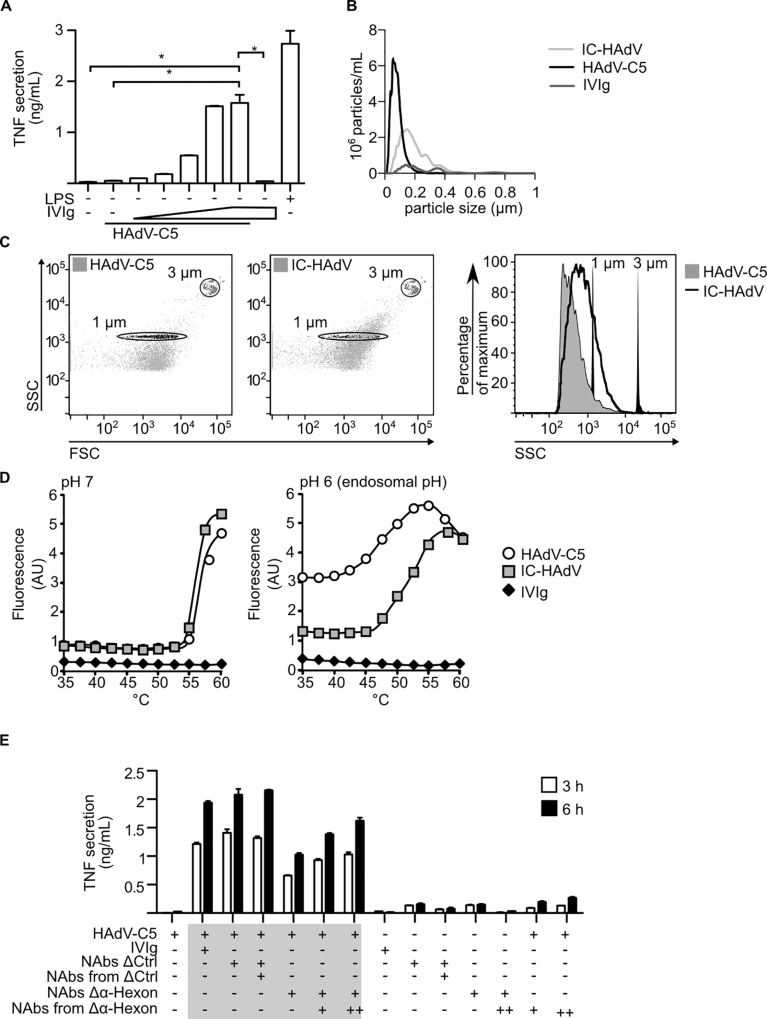
Immune-complexed HAdV-C5: role of IgGs, physical characteristics, and anti-hexon Abs. The effect of IgG-opsonization on HAdV-C5 particle size, stability, and aggregation, and how it affects MoDC maturation was assessed. A) TNF secretion by MoDCs incubated with LPS, HAdV-C5, IVIg and IC-HAdV formed using a fixed dose of 20,000 pp/cell HAdV-C5 and increasing volume of pooled human IgGs (IVIg). B) Size of HAdV-C5, IC-HAdV and IVIg was assayed by nanoparticle tracking. IC-HAdV were formed in PBS for 2 h at 37°C. C) Size of IC-HAdV-555 by flow cytometry. Fluorescent beads of 1 and 3 μm were used to determine the diameter of IC-HAdV based on side scatter (SSC). D) Fluorescent intensity used as a surrogate for capsid stability: the stability of HAdV-C5 or IC-HAdV was assessed at pH 6 and 7 at increasing temperatures by monitoring HAdV-C5 genome accessibility to Picogreen. E) Immune complexes were generated using either IVIg, mock-depleted IVIg (NAbs ΔCtrl), IgG recovered from mock-depleted IVIg (NAbs from ΔCtrl), from IVIg depleted for hexon-antibodies (NAbs Δα-hexon), or purified anti-hexon Abs (NAbs from Δα-hexon). MoDCs were incubated with HAdV-C5, IC-HAdV, IVIg for 3 or 6 h. TNF secretion was quantified by ELISA. All assays were repeated at least three times with similar results.

We previously showed that when HAdV-C5 is pre-incubated with human serum containing HAdV-C5 NAbs, the mix efficiently induce Fcγ-receptor (FcγR)-dependent MoDC maturation [36]. Previous reports also demonstrate that human sera or an adaptor made up of the extracellular fragment of the coxsackievirus and adenovirus receptor (CAR [59,60]) and the Fc portion of a human IgG increase HAdV-C5 internalization by FcγR-expressing cells [31,61]. Here, we compared the uptake of internalized capsids following incubation of HAdV-C5 or IC-HAdVs in MoDCs and found >10-fold more genomes internalized/cell at 6 h when using IC-HAdV (S1 Fig). These data are consistent with our previous studies using fluorescently labelled HAdV-C5 [62]. While MoDC maturation correlates with NAb titers, we could not formally exclude that other serum proteins influence MoDC maturation. To determine if IgG alone could induce MoDC maturation, we used pooled human IgGs (IVIg) to generate IC-HAdVs. In the presence of a fixed concentration of HAdV-C5 and increasing concentration of IVIg, MoDCs secrete increasing levels of TNF (Fig 1A). Neither HAdV-C5 nor IVIg induces significant levels of TNF after 6 h of incubation. Like our previous results using sera [36], IC-HAdVs made with IVIg induce higher levels of cell surface expression of co-stimulatory molecules CD40 and CD86 (S2A Fig). In addition, IC-HAdVs made with sera or IVIg induce a notable change in the size and granularity in ~40% of the cells (S2B Fig).

IC-HAdVs can be found in the systemic circulation during HAdV-C5 infection, and postmortem in tissues following fatal viremia [8,9]. Because the composition of IC-HAdVs could vary, we analyzed the size of IC-HAdV by flow cytometry and by nanoparticle tracking based on Brownian motion (NanoSight). Flow cytometry has a lower limit of ~1 μm, while NanoSight has an upper limit of ~1 μm in diameter. Using these complementary approaches, we found that most IC-HAdVs generated at 37°C for 2 h in PBS have a mean diameter of 200 nm prior to incubation with the cells (Fig 1B and 1C). Because an HAdV-C5 capsid is ~90 nm in diameter, these data suggest that in these conditions the majority of IC-HAdV have ~4 HAdV-C5 particles/complex.

Following engagement of receptor(s) on epithelial-like cells, HAdV-C5 is endocytosed in clathrin-coated pits. Then, HAdV-C5 escape from endocytic vesicles into the cytoplasm is concomitant with an acidification of the vesicles and prior to fusion with lysosomes [63]. The escape mechanism is thought to prevent HAdV-C5 from being degraded when the endocytic vesicles fuses with lysosome-like structures. By contrast, DCs delay acidification of some endocytic vesicles, which promotes more efficient antigen degradation for cross-presentation [64,65]. This is particularly efficient for IC antigens that are taken up by FcγRs [66–68]. Because HAdV-C5 alone poorly induce the maturation of MoDCs, it is likely that the NAb influence IC-HAdV trafficking. NAbs function differently depending on the targeted capsid protein. Anti-fiber NAb and anti-penton NAb likely impair cell surface receptor engagement [69], while hexon-NAb prevent extracellular recruitment of bridging molecules, intracellular capsid disassembly and/or recruitment of microtubule-associated motor proteins following endosome escape [70,71]. We therefore asked whether HAdV-C5 capsid stability changed following incubation with IVIg. To address capsid stability, we used accessibility of viral DNA to intercalating fluorescent dyes as a proxy to quantify capsid integrity/stability. Increased fluorescent intensity (decreased capsid integrity) was measured at pH 7 and 6 and at increasing temperature. At pH 7, we found similar kinetics for capsid disassembly for HAdV-C5 and IC-HAdV (Fig 1D, left panel). At pH 6, the fluorescence is higher for HAdV-C5 than for IC-HAdV throughout the temperature change below 55°C, indicating increased capsid stability in presence of NAbs (Fig 1D, right panel). Using chimeric HAdV capsids we previously showed that anti-hexon were responsible for the induction of DC maturation [72]. To address the role of anti-hexon Abs using another approach, we depleted them from IVIg and found that this decreases TNF secretion at 3 and 6 h postincubation (Fig 1E). Consistent with this, forming IC-HAdV with anti-hexon-Ab-depleted serum plus the respective eluate partly restored phenotype in a dose-dependent manner.

Together, these data demonstrate that MoDC maturation depends on IgG-HAdV-C5 complexes that contain ~4 HAdV-C5 capsids, increase uptake in MoDC, and the stabilizing anti-hexon Abs play a notable role in DC maturation.

### IC-HAdVs induce TNF production via TLR9 engagement

**Fig 2:**
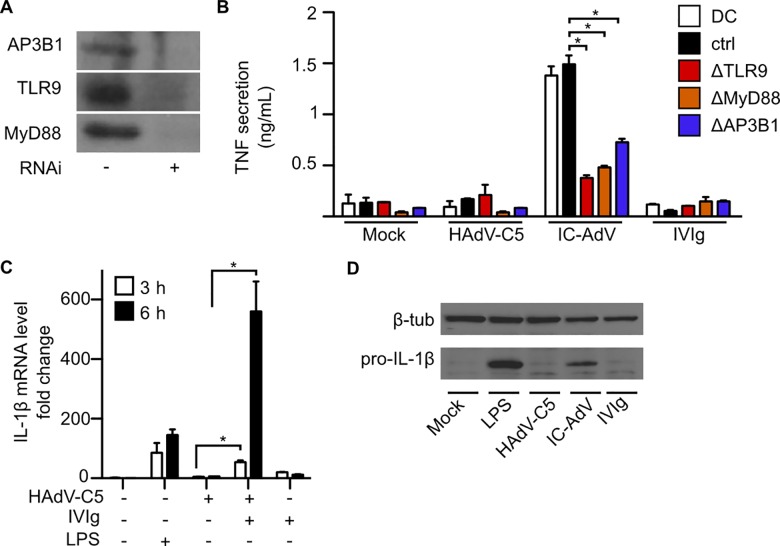
IC-HAdV induce MoDC maturation through DNA sensors TLR9. TNF secretion in response to IC-HAdV was measured in MoDCs after lentivirus-mediated shRNA knockdown of TLR9, MyD88 and AP3B1. A) Immunoblotting demonstrating lentivirus-mediated shRNA knockdown of the TLR9 pathway in MoDC. MoDCs were exposed to LPS, HAdV-C5, IC-HAdV or IVIg for 3 and/or 6 h and B) TNF secretion was measured by ELISA. C) IL1B mRNA levels were assessed by qRT-PCR. D) Immunoblots of pro-IL-1β levels in MoDCs after incubation with HAdV-C5, IC-HAdV, IVIg or LPS at 6 h. β-tubulin levels were used as loading controls. The experiments were carried out in 2–3 donors with similar results.

During FcγR-mediated uptake of immune-complexed cargo, TLR9 is also processed and delivered to lysosome-like compartments [73]. We reasoned that if NAb stabilize the HAdV-C5 capsid this should favor fusion of IC-HAdV-containing vesicles with TLR9^+^ vesicles and the induction of transcription of pro-inflammatory cytokines. Of note, using a TLR9 antagonist (IRS 869) we previously showed that IC-HAdV engage TLR9 [36]. Because pharmacological approaches can have off-target effects, we addressed the role of TLR9, its signaling adapter MyD88, and AP3B1, which is involved in TLR9 targeting to lysosome-related vesicles [74], by lentivirus-mediated shRNA knockdown. To circumvent SAMHD1 (sterile alpha-motif domain and His-Asp domain-containing protein 1)-mediated restriction of lentivirus infection of human monocytes, we generated lentivirus particles containing Vpx [54,75]. We also transduced freshly isolated monocytes prior to differentiation into DCs. Using this approach, ~90% of the monocytes are routinely transduced with the lentivirus vectors without inducing or preventing MoDC maturation. Although shRNA-mediated knockdown of TLR9, MyD88 and AP3B1 were partial (Fig 2A), TNF secretion is reduced in TLR9-pathway impaired cells (Fig 2B). We previously showed that IC-HAdV induce accumulation of *IL1B* mRNA in unprimed THP-1-derived DC [62]. We therefore asked whether IC-HAdV induce *IL1B* transcription in MoDC. We quantified *IL1B* mRNA levels by qRT-PCR at 3 and 6 h postincubation (Fig 2C). We found that MoDCs contain relatively low levels of *IL1B* mRNA with this classic differentiation protocol, while both LPS and IC-HAdV induce *IL1B* transcription and pro-IL-1β production (Fig 2D).

These data are consistent with our previous results [36] demonstrating that the HAdV genome is detected by the vesicular PRR TLR9, and induce production of pro-inflammatory cytokines.

### IC-HAdVs reach the cytoplasm

**Fig 3:**
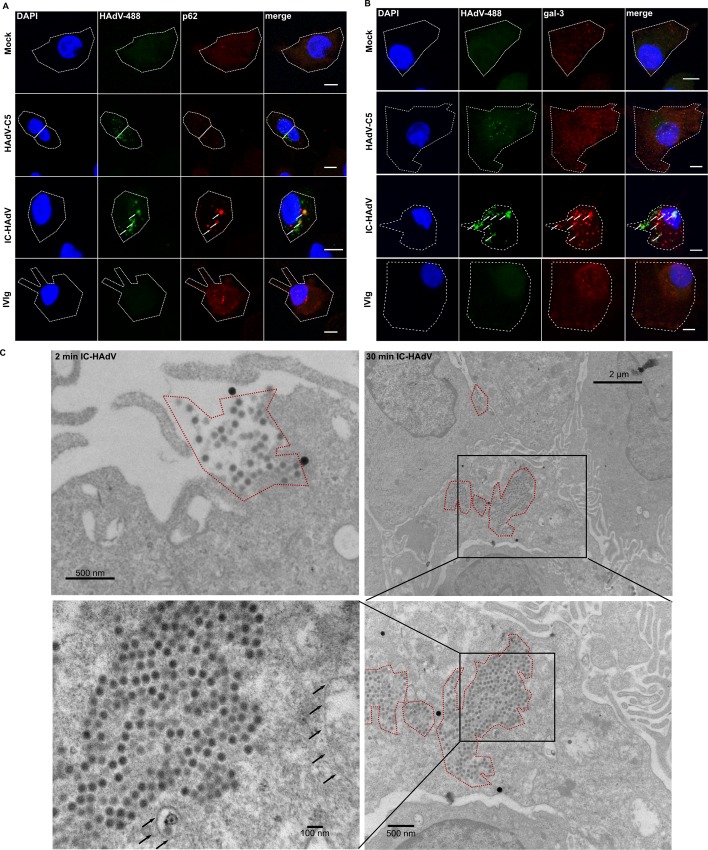
IC-HAdV induce membrane damage and gain access to the cytosol. MoDCs were exposed to Alexa488-HAdV-C5, IC-HAd-488 or IVIg for 3 h and processed for confocal immunofluorescence analysis and stained with anti-p62 (n = 3) A) and galectin-3 (n = 3) B). Scale bar = 5 μm. C) Transmission electron microscopy of IC-HAdV in MoDCs at indicated times. HAdV-C5 can be seen due to their high electron density. White lines depict circumference of IC-HAdVs. White asterisks indicate artifacts.

The above data point towards a process where HAdV-C5 capsids are disassembled in TLR9^+^ compartments, which should lead to protein VI release and disruption of the membrane [76]. We therefore used confocal microscopy to determine whether IC-HAdV induce membrane rupture. We focused on colocalization of Alexa488-labeled HAdV-C5 (HAdV-488) and IC-HAdV-488 with p62/sqstm1 and galectin-3. p62/sqstm1 is recruited to membrane remnants as part of the intracellular damage response and targets these remnants via binding to galectin-3 [77]. We found that p62/sqstm1 (Fig 3A) and galectin-3 (Fig 3B) colocalize with large IC-HAdV aggregates at 3 h postincubation, demonstrating disrupted membranes. We then used transmission electron microscopy (TEM) to visualize IC-HAdV at 30 min postincubation. In contrast to the ~200 nm aggregates detected in solution, we detect larger aggregates being internalized, and inside MoDCs (Fig 3C). Consistent with previous reports [61], the internalized of IC-HAdVs are not enclosed by membranes (Fig 3C). Together, these data demonstrate that IC-HAdVs begin to disassemble in TLR9^+^ endocytic vesicles, rupture their membranes, and escape into the cytoplasm.

### IC-HAdV-induced loss of cell membrane integrity

**Fig 4:**
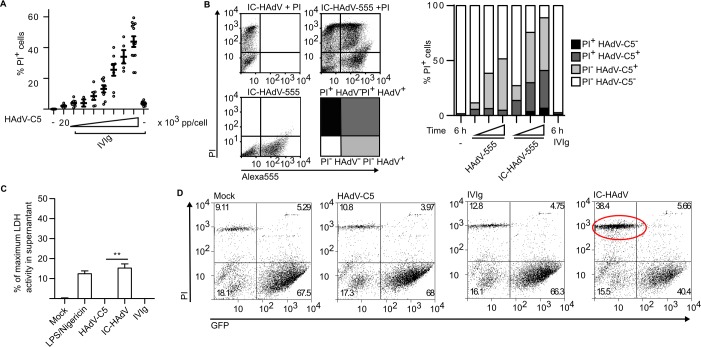
IC-HAdV induce loss of plasma membrane integrity and release of cytosolic proteins from MoDC. MoDCs were challenged with IC-HAdV and plasma membrane integrity, LDH and GFP release was quantified. A) MoDCs were incubated with IVIg, HAdV-C5 and IC prepared with an escalating dose (100, 1,000, 2,000 5,000, 10,000 15,000, 20,000 pp/cell) of HAdV-C5 and cell membrane integrity was assessed by intercalation of propidium iodide (PI) into cellular DNA at 6 h. B) MoDCs were incubated with Alexa555-HAdV-C5, IC-HAdV-555 or IVIg and assayed by flow cytometry at 6 h. The quadrants were set for PI^-^ HAdV-C5^-^ (white), PI^-^ HAdV-C5^+^ (light grey), PI^+^ HAdV-C5^+^ (dark grey) and PI^+^ HAdV-C5^-^ (black). The percentage of each subpopulation at each condition is depicted in the histogram. These assays were performed in 3 donors with similar results. C) Loss of cytosolic content by MoDC exposed to IC-HAdV, IVIg, HAdV-C5 and LPS/nigericin was assessed by measuring LDH activity in the supernatant. D) Loss of cytosolic content by GFP-lentivirus transduced MoDC exposed to IC-HAdV, IVIg, HAdV-C5 and LPS/nigericin was assessed by flow cytometry measuring GFP fluorescent and plasma membrane integrity with PI.

To further understand the IC-HAdV-induced change in MoDCs size and granularity we assayed membrane homeostasis. Of note, PMA-differentiated THP-1 cells primed with PAM3CSK4, a TLR1/2 agonist, and then exposed to 200,000 HAdV-C5 pp/cell lose plasma membrane integrity [28,78]. To determine if IC-HAdVs induce the loss of plasma membrane integrity we compared the effect of HAdV-C5 and IC-HAdV on MoDC. As markers for loss of membrane integrity we quantified propidium iodide (PI)^+^ entry into cells by flow cytometry and loss of intracellular proteins. Using a dose-dependent assay, we found that IC-HAdV made with as little as 5,000 HAdV pp/cell start to induce an increase in the number of PI^+^ cells at 6 h, which reached ~40% of the cells when using 20,000 HAdV pp/cell in this donor (Fig 4A). IC-HAdV-induced loss of plasma membrane integrity could be due to intrinsic or extrinsic factors. HAdV-C5 super-infection may induce loss of membrane integrity via intracellular ROS production and cathepsin release into the cytosol [28,78]. In addition, infection of mouse cells with HAdV-C5 leads to the release of cellular components (e.g. ATP), which are associated with the death of bystander cells [79,80]. To determine if IC-HAdV-mediated MoDC death is associated with direct interaction with IC-HAdV or a paracrine effect, we assayed MoDC interaction with IC-HAdV-555, an immune complex made using HAdV-555 (an Alexa555-labeled HAdV-C5). We found that all PI^+^ MoDCs are associated with IC-HAdV-555 (Fig 4B), suggesting that a direct interaction is responsible for the loss of plasma membrane integrity. These data demonstrate that PI, a small membrane impermeable DNA intercalator can enter IC-HAdV-challenged MoDCs.

To determine if the MoDC also lose intracellular proteins, we assayed extracellular levels of L-lactate dehydrogenase (LDH) activity and the loss of intracellular GFP in MoDC. We compared the maximum LDH activity in the supernatant of control and IC-HAdV-challenged MoDCs and found that IC-HAdV induce a level similar to LPS/nigericin (Fig 4C). To address this using another approach we transduced monocytes with a lentivirus vector expressing GFP, differentiated them into MoDCs, and challenged them with IC-HAdVs. We found that neither IVIg or HAdV-C5 significantly decreased the percentage of GFP+ MoDCs, while incubation with IC-HAdVs reduced the GFP population by ~40% (Fig 4D). Together, these data demonstrate that IC-HAdV-induced loss of cell membrane integrity.

### Protein VI and the HAdV genome are involved in loss of cell membrane integrity

**Fig 5:**
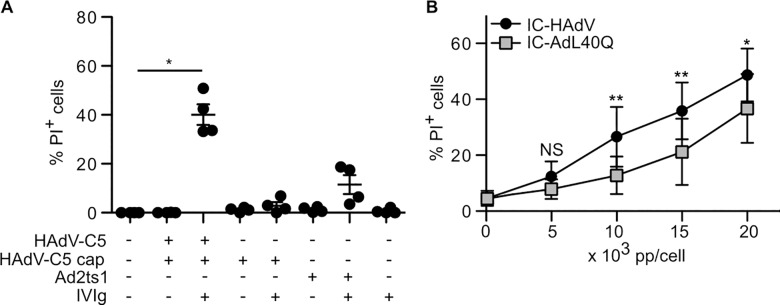
Protein VI is indirectly linked to loss of plasma membrane integrity. MoDCs were challenged with IC prepared from HAdV-C5 and HAdV-C5 harboring mutations in protein VI or protease. A) Effect on cell membrane integrity was determined by incubating MoDCs with HAdV-C5, Ad2ts1 and HAdV-C5 empty capsid or IC of each virus, respectively (n = 3) for 6 h, then with PI followed by flow cytometry. B) Dose-dependent loss of cell membrane integrity was assessed for IC-HAdV and IC-AdL40Q at 6 h (n = 5) as in B. Statistical significance (*p* values) were derived from two-way ANOVA with Bonferonni post test: * and ** correspond to p < 0.05 and p < 0.01, respectively.

To address the cause of IC-HAdV-mediated membrane permeabilization, we initially focused on the capsid components that likely influence IC-HAdV-mediated MoDC death. Endosomal escape of HAdV-C5 and induction of the NLRP3-inflammasome are linked in THP-1 cells [28,78] and therefore a similar mechanism might occur in primary human APCs. To address this possibility, we used Ad2ts1, which harbors a mutation in protease and results in a hyper-stable capsid [47,48]. In contrast to IC-HAdV, IC-empty capsids do not induce loss of membrane integrity, while IC-Ad2ts1 induce ~4-fold less (Fig 5A), suggesting that internal capsid components are linked to loss of membrane integrity.

We then focused on the role of the internal capsid protein VI. HAdV-C5 endosomal escape in epithelial cells depends on the membrane lytic capacity of protein VI [81]. Using AdL40Q, a HAdV-C5 vector containing a L40Q mutation in protein VI that causes attenuation in protein VI membrane lytic activity [45,46,82] we compared plasma membrane integrity in presence of IC-AdL40Q versus IC-HAdV in a dose-dependent assay. We found that IC-AdL40Q induces less plasma membrane disruption (Fig 5B) compared to IC-HAdVs. These data demonstrate that the membrane lytic activity of protein VI and the viral genome influence plasma membrane homeostasis.

### IC-HAdVs induce and colocalize with AIM2 inflammasomes

Our data indicate that IC-HAdV traffic through endosomal compartments until they fuse with a TLR9^+^ vesicle. DNA and protein VI are likely release from the capsid here, leading to TLR9 signaling and escape into the cytosol to induce viral genome-dependent loss of membrane integrity. Pioneering work by Wiethoff and colleagues showed that HAdV-C5 can activate the NLRP3 inflammasome in super-infected THP-1 cells, and in human monocyte-derived macrophages [78]. We therefore asked whether the NLRP3 pathway is being activated in IC-HAdV-challenged MoDCs. Although the mechanism is poorly understood, the NLRP3-mediated inflammasome activation is critically dependent on K^+^ efflux. We therefore increased the extracellular concentration of K^+^ to block the NLRP3 pathway and found that this had no effect on the loss of MoDC membrane integrity upon challenge with IC-HAdV (S3A and S3B Fig). Similarly, MCC950, an inhibitor of canonical and noncanonical NLRP3 activation did not prevent the loss of membrane integrity (S3C Fig). From these data we concluded that the NLRP3 inflammasome was not activated in unprimed human MoDCs.

**Fig 6:**
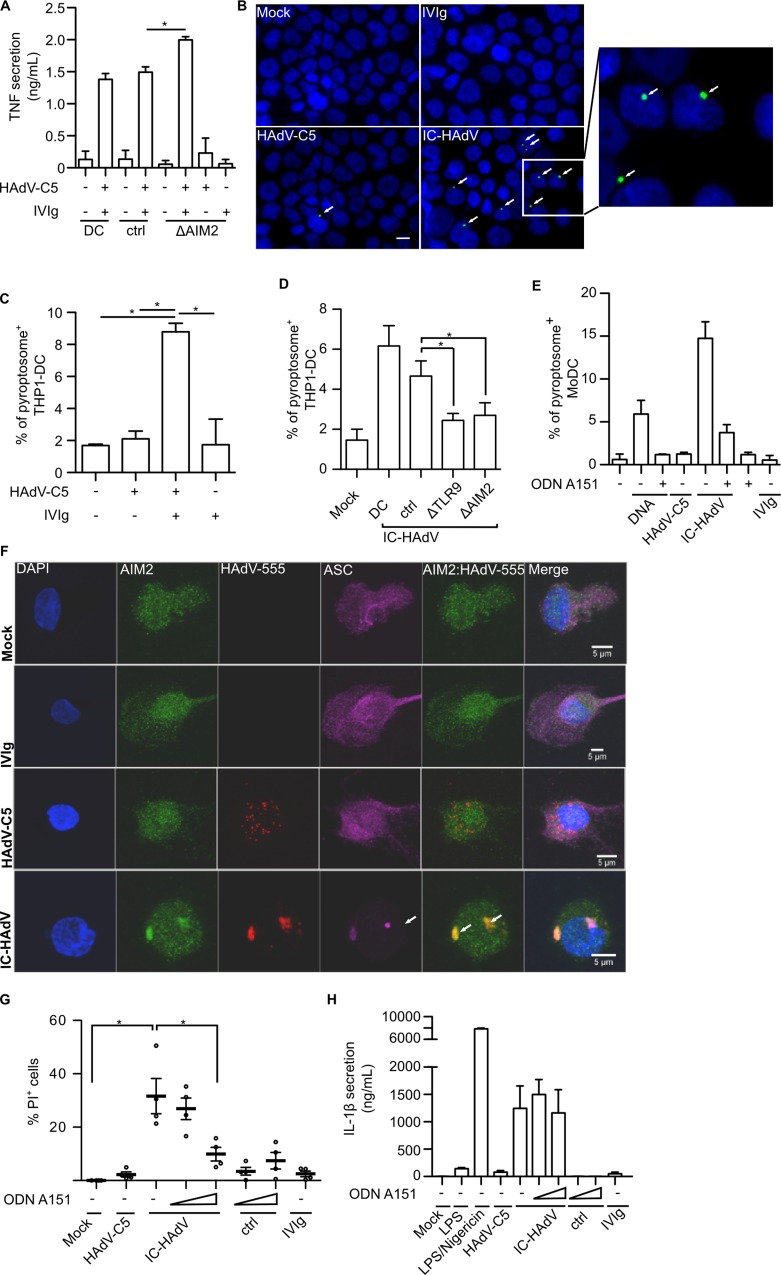
IC-HAdVs induce AIM2-associated pyroptosomes. Involvement of AIM2 and/or TLR9 in IC-HAdV-induced inflammatory cell death was assessed by RT-qPCR, RNAi, pharmacological inhibitors, flow cytometry, fluorescence microscopy and ELISA. A) Lentiviral-mediated shRNA knockdown of AIM2 in MoDC. MoDCs were exposed to HAdV-C5, IC-HAdV or IVIg for 6 h and TNF secretion was quantified by ELISA. B) Fluorescent microscopy of THP-1 ASC-GFP-derived dendritic cells incubated with HAdV-C5, IC-HAdV or IVIg. White arrows indicate ASC pyroptosomes (green). Nuclei were counter stained with DAPI (blue). Scale bar = 20 μm. C) Percentage of pyroptosome formation in THP-1 ASC-GFP-derived dendritic cells 1 h poststimulation. D) Percentage of pyroptosome formation in THP-1 ASC-GFP-derived dendritic cells after 1 h exposure to IC-HAdV after shRNA knockdown of MyD88, TLR9 and AIM2. E) Percentage of pyroptosomes (immune-labeled with anti-ASC) in MoDC ± AIM2-inhibitor ODN A151 for 2 h and exposure to transfected DNA, IVIg, HAdV-C5 and IC-HAdV for 3 h. F) MoDCs were exposed to Alexa555-HAdV-C5, IC-HAdV-555 or IVIg for 3 h and processed for confocal immunofluorescence analysis and stained with anti-AIM2 and anti-ASC. Nuclei were counter stained with DAPI (blue) (n = 3). Scale bar = 5 μm. G) Loss of plasma membrane integrity was assessed by PI/flow cytometry in ODN A151-pretreated MoDCs challenged with IC-HAdVs. MoDCs were exposed to 20,000 pp/cell HAdV-C5, IC-HAdV or IVIg after 2 h pre-incubation with 10 or 100 μM ODN A151 H) Conditions as in G, IL-1β secretion was quantified by ELISA at 6 h (n = 3).

In addition to the NLRP3, inflammasome formation can be induced by AIM2, IFI16, NLRP1, NLRC4 pathways [83]. Because AIM2 detects foreign dsDNA, its potential role in IC-HAdV-challenged MoDCs was investigated. Monocytes are classically differentiated into MoDCs in presence of GM-CSF and IL-4. Of note, IL-4 can inhibit IL-1β secretion and NLRP3 inflammasome induction in a non-transcriptional manner [84,85]. Moreover, compared to monocytes, MoDCs secret notably less IL-1β in response to TLR2/4 agonists and heat-killed bacteria [86]. By contrast, IL-4 does not affect the AIM2 inflammasome components [85], and cytosolic DNA stimulates upregulation of *AIM2* mRNA levels and AIM2 inflammasome activation in MoDCs [87]. We therefore quantified *NLRP3* and *AIM2* mRNA levels in freshly purified monocytes, immature MoDCs, and in MoDCs incubated with IC-HAdV or LPS. We found that in MoDCs, *NLRP3* mRNA levels are approximately 10-fold lower than in monocytes and, in contrast to LPS and IC-HAdV, do not increase *NLRP3* mRNA levels (S4A Fig). By contrast, *AIM2* mRNA levels are modestly increased during monocytes differentiation into MoDC, and are upregulated in response to LPS and IC-HAdVs (S4B Fig). We therefore used the lentivirus shRNA approach to reduce AIM2 levels in MoDCs (S4C Fig). Following AIM2 reduction and IC-HAdV challenge, we found that TNF levels increase compared to controls (Fig 6A). These data suggest an impaired AIM2 inflammasome formation, prolonged survival of MoDC, and a continued production and secretion of TNF stimulated by upstream TLR9 engagement.

The assembly of the AIM2 inflammasome is a two-step process. First, several AIM2 molecules bind to DNA via the C-terminal HIN200-domain and oligomerize through protein-protein of the respective pyrin domains [88]. The ability to oligomerize is critical for dsDNA binding and the length of DNA regulates the assembly of the AIM2 polymers. Second, AIM2 oligomerization leads to the recruitment of ASC, caspase 1, pro-IL-1β, and GSDMD. To determine if IC-HAdVs induce inflammasome formation, we used THP-1-derived DC expressing an ASC-GFP fusion protein (THP-1-ASC-GFP [41]) to visualize inflammasome formation. Under mock-treated conditions, ASC-GFP is dispersed throughout the cytoplasm (Fig 6B, top left panel). After inflammasome assembly a GFP foci/cell can be detected [41]. We therefore incubated THP-1-ASC-GFP DC with IC-HAdVs and quantified pyroptosome formation. We found that IC-HAdVs induce >5-fold more (*p* < 0.05) inflammasomes than in mock-, HAdV-C5-, or IVIg-challenged THP-1 cells (Fig 6C and 6D). We then repeated the shRNA-mediated knockdown of AIM2 in THP-1-ASC-GFP cells. Knockdown of AIM2 reduces inflammasome formation in response to IC-HAdVs (Fig 6D). Interestingly, knockdown of TLR9 also reduces AIM2-associated inflammasome formation also (Fig 6D) in THP-1 cells. These data suggest that the trafficking of the IC-HAdV through a TLR9^+^ vesicle may increase the expression of AIM2 inflammasome components and/or formation.

Because in our hands in THP-1-ASC-GFP cells responded poorly to IC-HAdV challenge, we also assayed pyroptosome formation in MoDCs. Here we found that IC-HAdVs induced at least ~15-fold more pyroptosome^+^ MoDCs than controls (Fig 6E). Of note, these results likely underestimate the percentage of MoDCs with pyroptosomes: there were consistently fewer intact cells bound to the poly-L-lysine-coated coverslips capable to be analyzed by immunofluorescence staining for ASC aggregates.

We then asked if pyroptosome formation is directly associated with the IC-HAdVs in MoDCs. To address this question, we incubated MoDCs with IC-HAdV-555 and screened for AIM2 and ASC subcellular location by epifluorescence and immunofluorescence. Consistent with our previous results, IVIg and Alexa555-HAdV-C5 alone do not induce inflammasome formation (Fig 6F). By contrast, as early as 30 min postincubation AIM2 and ASC colocalized with IC-HAdV-555 in large fluorescent aggregates (Fig 6F). To address AIM2 inflammasome formation using another approach we pre-incubated cells with ODN A151, a competitive inhibitor for DNA recognition by AIM2. ODN A151 can prevent AIM2-induced inflammasome assembly, caspase 1 induction, and IL-1β maturation in APCs [50]. Following pre-incubation of MoDCs with ODN A151 we found a dose-dependent inhibition of IC-HAdV-induced loss of cell membrane integrity (Fig 6G). Importantly, pre-incubation with ODN A151 also rescued MoDCs from loss of cell membrane integrity when cells are transfected with plasmid DNA (S5 Fig). Unexpectedly, ODN A151 did not efficiently prevent IC-HAdV IL-1β secretion (Fig 6H). These data indicate that inhibition of cytosolic DNA sensing by AIM2 prevents IC-HAdV-induced loss of cell membrane integrity, but not the release of IL-1β. Together, these data demonstrate that IC-HAdV induce AIM2/ASC aggregates in human MoDCs.

### IC-HAdVs induce pyroptotic MoDC death via the AIM2 inflammasome

**Fig 7:**
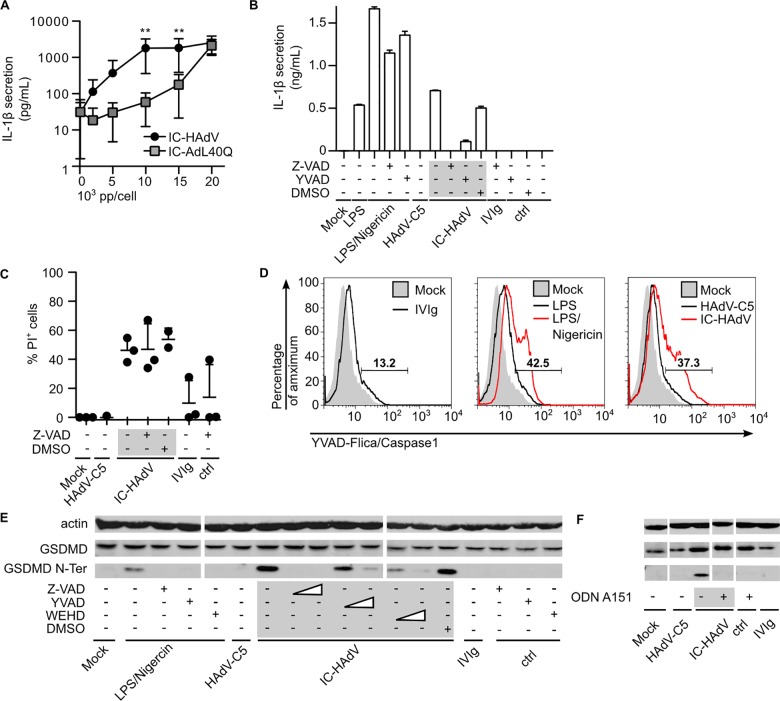
IC-HAdV induced IL-1β expression, caspase-dependent IL-1β secretion and AIM2 inflammasome-dependent GSDMD cleavage in MoDCs. The involvement of the AIM2 inflammasome and caspases in the inflammatory response in IC-HAdV challenged MoDCs ± inhibitors was assessed by quantifying intra- and extracellular levels of IL-1β plasma membrane integrity and GSDMD cleavage. A) Effect of protein VI-mediated membrane lytic activity on IL-1β secretion was assessed by incubation of MoDCs with escalating doses of IC-HAdV or IC-AdL40Q. IL-1β secretion was quantified by ELISA (n = 5). P values were derived from two-way ANOVA with Bonferroni posttest. * and ** correspond to p < 0.05 and p < 0.01, respectively. B) IL-1β secretion in MoDCs pre-incubated with 20 μM YVAD and Z-VAD and stimulated as indicated. This experiment was performed in triplicate using 3 donors with similar results. C) Loss of cell membrane integrity was assessed by PI/flow cytometry for MoDCs pre-incubated with Z-VAD and exposure to HAdV-C5, IC-HAdV and IVIg at 6 h (n = 3). D) Caspase 1 activation was measured with FAM-YVAD-FMK FLICA by flow cytometry. MoDC were preincubated with YVAD-FLICA for 1 h and exposed to LPS, LPS/nigericin, IVIg HAdV-C5 and IC-HAdV for 3 h. Nigericin was added to MoDC at 2 h. This assay was performed in 3 donors with similar results E) GSDMD cleavage in IC-HAdV-challenged MoDC in presence of caspase inhibitors was monitored by western blot. MoDC were preincubated for 1 h with 20 or 100 μM Z-VAD, YVAD, WEHD or an equal volume of DMSO followed by exposure to LPS/nigericin (added at 4 h), IVIg, HAdV-C5 and IC-HAdV for 6 h. This assay was performed in 3 donors with similar results. F) GSDMD cleavage in IC-HAdV-challenged MoDC in presence of AIM2 inhibitors was monitored by immunoblotting. MoDC were pre-incubated for 2 h with 100 μM ODN A151 followed by exposure to IVIg, HAdV-C5 and IC-HAdV for 6 h This assay was performed in 3 donors with similar results.

Pyroptosis is a highly inflammatory form of cell death. The key determinants for pyroptosis are pyroptosome formation (Fig 6), caspase 1 activation, IL-1β secretion, and loss of cell membrane integrity (Figs 4 and 5) due to cleavage of GSDMD. Canonically, IL-1β is cleaved by auto-activated human caspases 1 upon inflammasome formation. To address whether IL-1β is secreted we incubated MoDCs with IC-HAdV and quantified IL-1β levels in the supernatant. We found that IC-HAdV induce a dose-dependent increase in secreted IL-1β levels (Fig 7A). Due to our results demonstrating that IC-AdL40Q induces less plasma membrane disruption, we also quantified the level of secreted IL-1β from IC-HAdV- and IC-AdL40Q-challenged MoDCs. In these assays, we found less secreted IL-1β-induced by IC-AdL40Q at lower doses (Fig 7A). These data suggest that endosomal escape is needed for the secretion of IL-1β.

The reports describing the involvement of caspase 1 in HAdV-C5-induced cell death vary. While both HAdV-C5 and IC-HAdV induce caspase 1 activation and IL-1β processing in THP-1 cells [30], the caspase 1 inhibitor YVAD does not inhibit the loss of membrane integrity [78]. We therefore addressed the role of caspases in IC-HAdV-challenged MoDC. We incubated cells with a pan-caspase inhibitor (Z-VAD) and an inhibitor for caspase 1 (YVAD) prior to a challenge with IC-HAdVs. We found that both inhibitors significantly reduced the levels of secreted IL-1β (Fig 7B), demonstrating that IC-HAdV induce caspase 1 activation. Of note, Z-VAD did not prevent PI from entering cells (Fig 7C), suggesting that a caspase-independent factor may be causing small holes in the plasma membrane. To address caspase 1 activation using another approach, we incubated cells with the fluorescent probe FAM-YVAD-FMK FLICA, which enters each cell and irreversibly and covalently binds to activated caspase 1. We quantified the fluorescent cells by flow cytometry and found that ~40% of the cells contain activated caspase 1 (Fig 7D). Together, these data demonstrate that IC-HAdVs induce caspase 1 activation and IL-1β release.

Like caspase 1, GSDMD is recruited with similar kinetics and in similar amounts to pyroptosomes [17]. To determine if IC-HAdV-challenge induces GSDMD cleavage we assayed GSDMD cleavage by immunoblotting. We found that GSDMD is efficiently cleaved in IC-HAdV-challenged MoDCs, and cleavage could be prevented by caspase 1-specific inhibitors (YVAD and WEHD) as well as Z-VAD (Fig 7E). In addition, ODN A151, which inhibits AIM2 engagement of dsDNA, also reduced GSDMD processing (Fig 7F). Together, these data demonstrate that IC-HAdVs induce the key determinants of pyroptosis in MoDCs.

## Discussion

**Fig 8:**
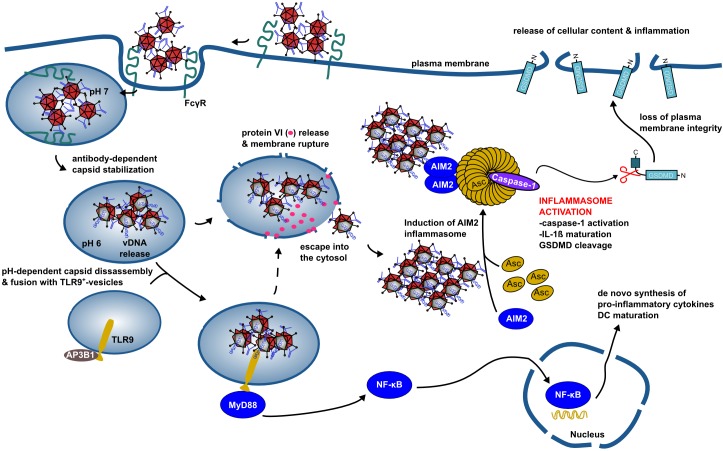
IC-HAdV trafficking and cellular events leading to pyroptotic dendritic cell death. IC-HAdV are taken up via FcγR-mediated endocytosis and fuse with TLR9^+^ endolysosomal vesicles because of NAb-dependent capsid stabilization at endosomal pH. At the more acidic pH in lysosome-like vesicles, IC-HAdV begin to disassemble and to release viral DNA, membrane lytic protein VI, and cellular components. TLR9 engagement induces MyD88-NF-κB-dependent *de novo* expression of TNF and pro-IL1β. During vesicular DNA sensing, protein VI release from the capsid leads to endolysosomal membrane lysis and IC-HAdV gain access to the cytosol. The cytosolic inflammasome sensor AIM2 interacts with the HAdV-C5 genome and induces inflammasome assembly and activation of caspase 1, and downstream cleavage of pro-IL-1β and GSDMD. Together this leads to pyroptosis of MoDCs.

The aim of our study was to understand the impact of immune-complexed HAdV-C5 on human APCs. We show that anti-HAdV-C5 hexon Abs aggregate and stabilized the capsid at mildly acidic pH. The anti-hexon antibodies also perturb the intracellular trafficking of HAdV-C5: instead of release from early endocytic vesicles, the IC-HAdV^+^-vesicles fuse with TLR9^+^ vesicles. Here, the capsid must be partially disassembled to allow detection of the genome by TLR9. TLR9 engagement induces the transcriptional activation of pro-inflammatory cytokines and inflammasomes components via the MyD88-NF-κB signaling pathways. Concomitant with genome release from the capsid, the membrane lytic activity of protein VI impacts IC processing by permitting endocytic vesicle cargo escape into the cytoplasm. Here, the 36 kb dsDNA HAdV genome is detected by AIM2, which initiates the nucleation of an inflammasome. Recruitment and cleavage of caspase 1, pro-IL-1β and GSDMD culminate in pyroptotic MoDC death (Fig 8).

### The ex vivo human APC model

Monocytes are an evolutionarily conserved subset of highly phagocytic mononuclear cells that originate from myeloid progenitors in the bone marrow. They represent 10% of the white blood cells, including reservoirs in the spleen and lungs. Monocytes are rapidly recruited to tissues during infection and inflammation. Here, after exposure to pro-inflammatory cytokines and microbial compounds they differentiate into macrophages or DCs, and then initiate antimicrobial activity or promote T-cell responses. These characteristics make MoDCs a powerful and clinically relevant *ex vivo* system to address the effect of human pathogens. An advantage when using primary cultures of monocytes is the real-life variations associated with donors, while a challenge is the generation of mutant cells that are defective in a specific pathway–without perturbing their capacity to be influenced by the stimuli. We addressed these challenges by repeating the assays in cells from multiple donors and by using lentivirus vector shRNA-mediated knockdown in freshly isolated monocytes prior to differentiation, respectively.

Previous studies addressing HAdV vector infection of THP-1 cells and murine macrophages reported the activation of the NLRP3 inflammasome [28,30,78]. Barlan *et al*. reported that HAdV-C5 can prime THP-1 cells for expression of pro-IL-1β and NLRP3 via TLR9 signaling. HAdV-C5 also induces the NLRP3 inflammasome in both PAM3CSK4-primed and unprimed THP-1 cells [78]. Notably, *NLRP3* mRNA levels are downregulated during the differentiation from monocytes into MoDC, while *AIM2* mRNA levels are not affected. In addition, IC-HAdV challenge increased *AIM2* mRNA, but not *NLRP3* mRNA levels in MoDC. In spite of the prevailing dogma that HAdV-C5 is highly immunogenic, we routinely find that compared to murine cells and human cell lines, HAdV-C5 internalization poorly induces human DC maturation [36]. Of note though, transfection of HAdV-C5 DNA into primary cultures of human keratinocytes induces an NLRP3 response [89]. Yet, our data argue against the involvement of NLRP3 in MoDCs challenged with IC-HAdVs. A significant difference in the induction of the NLRP3 and AIM2 inflammasome is NLRP3’s requirement for priming [90–93]. Canonically, NLRP3 activation is a two-step mechanism: initial activation includes the *de novo* expression of inflammasome components (including NLRP3), and a second stimulus triggers inflammasome assembly [91]. A fundamentally different complex is assembled by AIM2, because NLRP3 can self-oligomerize. AIM2 binds dsDNA at regular intervals in a length-dependent manner. The relatively long linker between the HIN and the pyrin domain would then allow the PYDs from several AIM2 molecules to swing around the DNA core and oligomerize, thereby nucleating ASC polymerization. The linear, 36-kb long HAdV genome likely makes it an ideal AIM2 target and, importantly, aggregated HAdV genomes can be easily detected in the cytoplasm of IC-HAdV-challenged MoDCs (S4D Fig).

Intuitively, there would appear to be a selective advantage for a host to be able to use AIM2 and NLRP3 in concert to fight pathogens [25,94]. In HeLa cells transfected with plasmids to overexpress TRIM21, this intracellular FcR detects IC-HAdV and to target them for proteasome degradation [95,96]. Because IC-HAdV access the cytosol in MoDC, it would have been interesting to determine if this degradation pathway for cytosolic immune complexes is physiologically relevant in bona fide human APCs. However, TRIM21 knockdown in MoDCs was inefficient in our hands.

### Pyroptosis: pro-virus or pro-host?

Whether IC-HAdV-induced pyroptosis in other FcγR^+^ cells (e.g. neutrophils) occurs in a host with HAdV NAbs during local or systemic injection of HAdV vectors is unknown. Intriguingly, neutropenia has been repeatedly associated with HAdV infections [97,98] and with HAdV vector injections [99]. Like for HAdV-C5, humoral immunity increases inflammasome induction for *Staphylococcus aureus* compared to the non-opsonized pathogen [100]. But, pyroptotic cells death is not a universal response to ICs: ICs containing ovalbumin, sheep red blood cells, or *Candida albicans* block activation and assembly of AIM2, NLRP3 or NLRC4 inflammasomes via ligation of activating FcγRs [101]. How ICs affect inflammasome induction likely depends on the size and topography of the complexes, the number of IgGs in the complex, its ability to cluster and signaling through the FcγRs, and the ability of the antigen/pathogen to influence its processing. Most experiments that quantify pathogen burden in the presence and absence of pro-inflammatory caspase-dependent immune responses suggest a protective effect. However, disease progression is neither linear, nor identical, in all environments. Some pathogens may benefit from the activation of pro-inflammatory response during specific stages of infection–in particular those that infect immune cells that are recruited to the site of inflammation. HIV-1 is an interesting example; it infects activated CD4^+^ T cells that are recruited to the site of infections, which results in an IFNγ-inducible protein 16-mediated pyroptosis of the bystander T cells [102].

For IC-HAdVs, opposing arguments for whether pyroptotic DC death is pro-virus or pro-host could be made. Because TLR engagement increases phagosome maturation, lysosomal acidification, and antigen degradation in murine DCs [103] rapid escape from an environment that promotes antigen presentation may preclude efficient memory T-cell stimulation and be pro-HAdV. Destroying DCs by pyroptosis would be an efficient mean to prevent the stimulation of memory T cells. However, pyroptosis is not an immunologically sterile response and should stimulate the infiltration of immune cells into the site of infection that should eventually lead to pathogen control. In a healthy host, recurrent HAdV infections are readily controlled, which suggests that this phenomenon should be, in most cases, pro-host. Yet, the equilibrium between IC-HAdV formation, MoDC maturation, T-cell activation, and pyroptotic DC death may depend on the dynamic HAdV load during the course of a *de novo* infection or immune escape. Indeed, we show that increased capsid stability is associated with greater TNF secretion. IC-induced pyroptosis of FcγR^+^ cells may become widespread during HAdV-disseminated disease in T-cell deficient hematopoietic stem cell transplant patients. In this regard, IC-HAdV likely form during HAdV infection because type-specific Abs are present in patients [6] and thus, may contribute to the adverse effects like inflammation and trigger or worsen graft-versus host disease [10–12,104]. Of note, inflammasomes can be ejected from cells, ingested by bystander cells, and amplify a proinflammatory response [105,106]. Whether this phenomenon plays a role in the IL-1β levels in the supernatant, even after blocking the majority of AIM2 inflammasomes with ODN A151, needs focused attention. Intriguingly, mucosal homing Th17 anti-HAdV T cells may be involved in acquisition of HIV infection following HAdV-based vaccination [37–39]. IL-17 secretion correlated with HAdV-C5 pre-existing immunity when PBMC from vaccinees in the STEP trial where re-stimulated with HAdV-C5 [107]. This implies that upstream of the Th17 T-cell differentiation IL-1β plays a role that may be linked to IC-HAdV-mediated pyroptosis.

In summary, our study provides a mechanistic understanding of how pre-existing humoral immunity to HAdVs impact innate immunity via PRR in *bona fide* human APCs. Understanding of the complex interplay between HAdV, NAbs, and innate sensor in human APCs may enable us to develop therapies to treat disseminated HAdV-disease, optimize AdV-based vaccines, and improve AdV-mediated gene transfer.

## Supporting Information

S1 Table Sequences or references (Open BioSystems) of the shRNAs used to knockdown the production of the indicated proteins. (DOCX)

S1 Fig IgG-opsonization of HAdV-C5 increases binding and internalization into MoDC. Internalization of HAdV-C5 ± IVIg was assessed by RT-qPCR the transgene and *GAPDH* in DNA extracts from the indicated times. For each time point, total versus intracellular virus has been distinguished by acid wash which partially removed extracellular capsid These assays were performed in triplicate with more than 3 donors with similar results.
(TIF)

S2 Fig IC-HAdV made using IVIg induce DC maturation. MoDCs were incubated with HAdV-C5, IC-HAdV, IVIg or LPS for 6 h. Flow cytometry profile of A) CD40 and B) CD86 in MoDCs treated with the different stimuli compared to mock-treated cells (grey). C) MoDC were exposed to HAdV-C5, IVIg and IC-HAdV for 30 min. Cell morphology was assayed by flow cytometry.
(TIF)

S3 Fig IC-HAdV do not induce the NLRP3 inflammasome. Involvement of NLRP3 in IC-HAdV-challenged was assessed by PI/flow cytometry. MoDC were preincubated with NLRP3-inhibitors KCl (20 and 40 mM) and 10 μM MCC950 for 1 h. or A) mock-treated or exposed to LPS/nigericin and B) 20 and 40 mM KCl C) 10 μM MCC950. These experiments were carried out in at least 2 individual donors with similar results.
(TIF)

S4 Fig Expression levels of inflammasome sensors. RT-qPCR analysis of A) *AIM2* B) and *NLRP3* mRNA levels in monocytes and MoDCs and after challenge with LPS or IC-HAdV in MoDC. These assays were performed in triplicate using 3 donors with similar results. C) Immunoblotting demonstrating lentivirus-mediated shRNA knockdown of AIM2 in MoDC. D) Viral DNA is readily detected in the cells and remains associated with viral capsid in IC-HAdV-challenged MoDC. MoDC were exposed to IC-HAdV-488 for 3 h and prepared for fluorescence microscopy with DAPI as counterstaining.
(TIF)

S5 Fig Plasmid DNA induces loss of membrane integrity. MoDCs were pre-incubated with 10, 50 or 100 μM ODN A151 for 2 h and transfected with plasmid DNA complexed by Lipofectamine LTX and cell membrane integrity was assessed by PI/flow cytometry (n = 2).
(TIF)
