# Supplementary material for: Immune-Complexed Adenovirus Induce AIM2-Mediated Pyroptosis in Human Dendritic Cells
Source: PLoS Pathog. 2016 Sep 16;12(9):e1005871. doi: 10.1371/journal.ppat.1005871 (PMC5026364; doi:10.1371/journal.ppat.1005871)
Supplement: S1 Table — (DOCX) [file ppat.1005871.s001.docx]

**Table S1) shRNA reference or sequence**

| Gene target | shRNA reference or sequence |
| --- | --- |
| TLR9 | TRCN0000056891  TRCN0000056892 |
| AP3B1 | TRCN0000065059  TRCN0000065060 |
| AIM2 | TRCN0000107503  TRCN0000107504 |
| MyD88 | CCGCCTGTCTCTGTTCTTGAACctgtgaagccacagatgggGTTCAAGAACAGAGACAGGCGG  TGGTGGTTGTCTCTGATGATTActgtgaagccacagatgggTAATCATCAGAGACAACCACCA |
