# Supplementary figures and images for: Immune-Complexed Adenovirus Induce AIM2-Mediated Pyroptosis in Human Dendritic Cells
Source: PLoS Pathog. 2016 Sep 16;12(9):e1005871. doi: 10.1371/journal.ppat.1005871 (PMC5026364; doi:10.1371/journal.ppat.1005871)

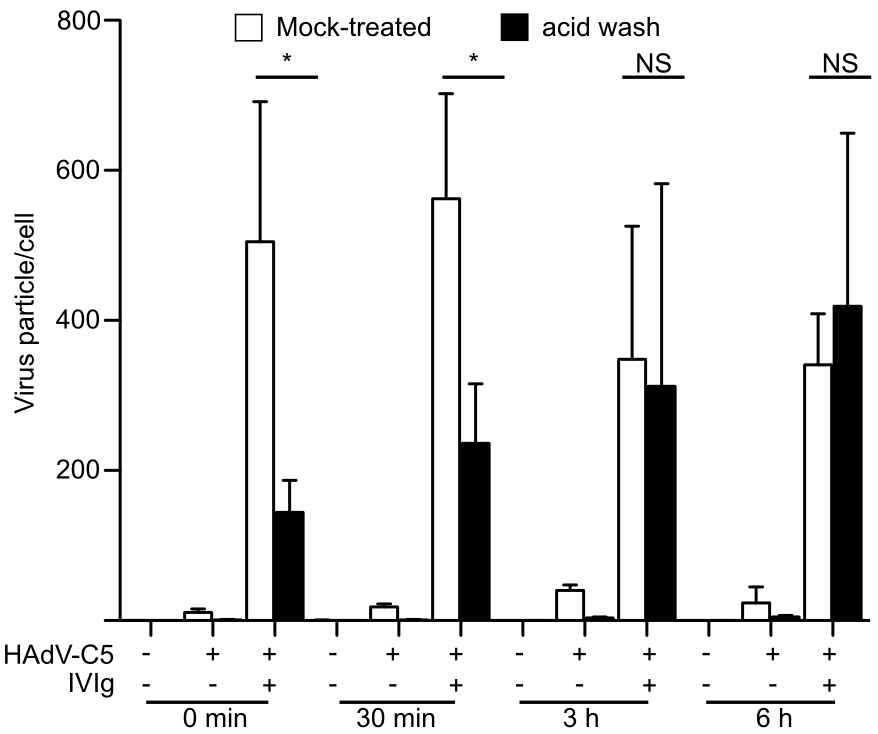

Supplement: S1 Fig — Internalization of HAdV-C5 ± IVIg was assessed by RT-qPCR the transgene and GAPDH in DNA extracts from the indicated times. For each time point, total versus intracellular virus has been distinguished by acid wash which partially removed extracellular capsid These assays were performed in triplicate with more than 3 donors with similar results. (TIF) [file ppat.1005871.s002.tif]

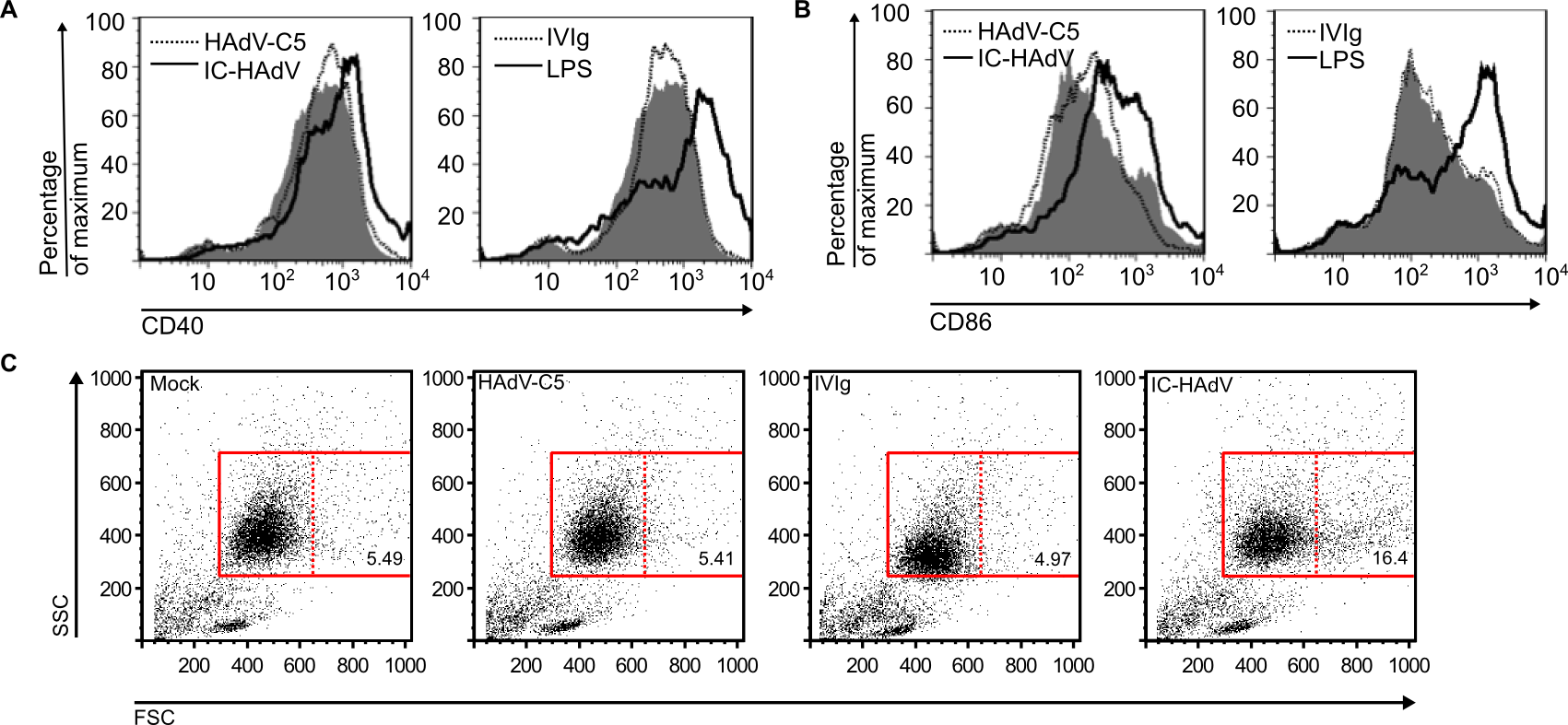

Supplement: S2 Fig — MoDCs were incubated with HAdV-C5, IC-HAdV, IVIg or LPS for 6 h. Flow cytometry profile of A) CD40 and B) CD86 in MoDCs treated with the different stimuli compared to mock-treated cells (grey). C) MoDC were exposed to HAdV-C5, IVIg and IC-HAdV for 30 min. Cell morphology was assayed by flow cytometry. (TIF) [file ppat.1005871.s003.tif]

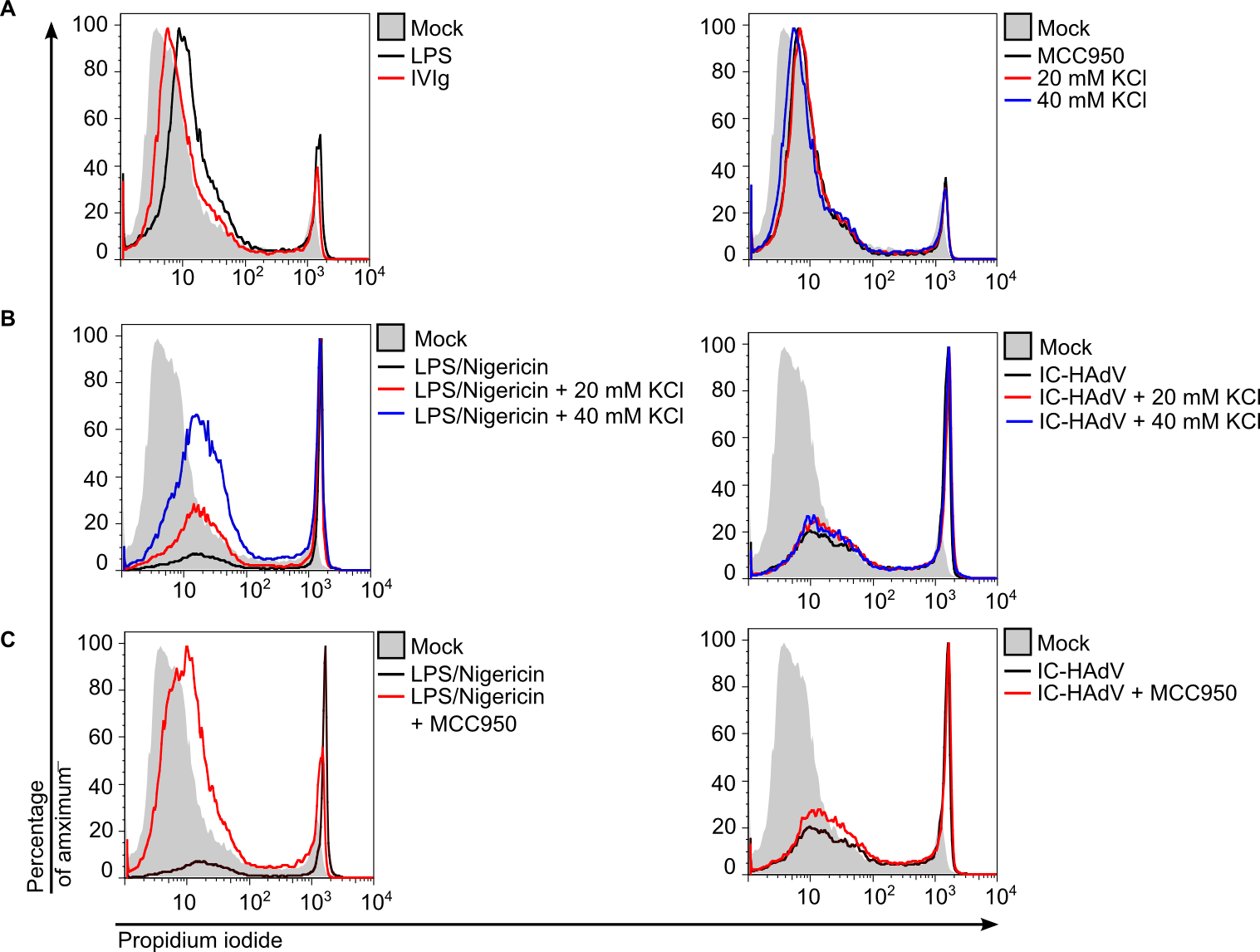

Supplement: S3 Fig — Involvement of NLRP3 in IC-HAdV-challenged was assessed by PI/flow cytometry. MoDC were preincubated with NLRP3-inhibitors KCl (20 and 40 mM) and 10 μM MCC950 for 1 h. or A) mock-treated or exposed to LPS/nigericin and B) 20 and 40 mM KCl C) 10 μM MCC950. These experiments were carried out in at least 2 individual donors with similar results. (TIF) [file ppat.1005871.s004.tif]

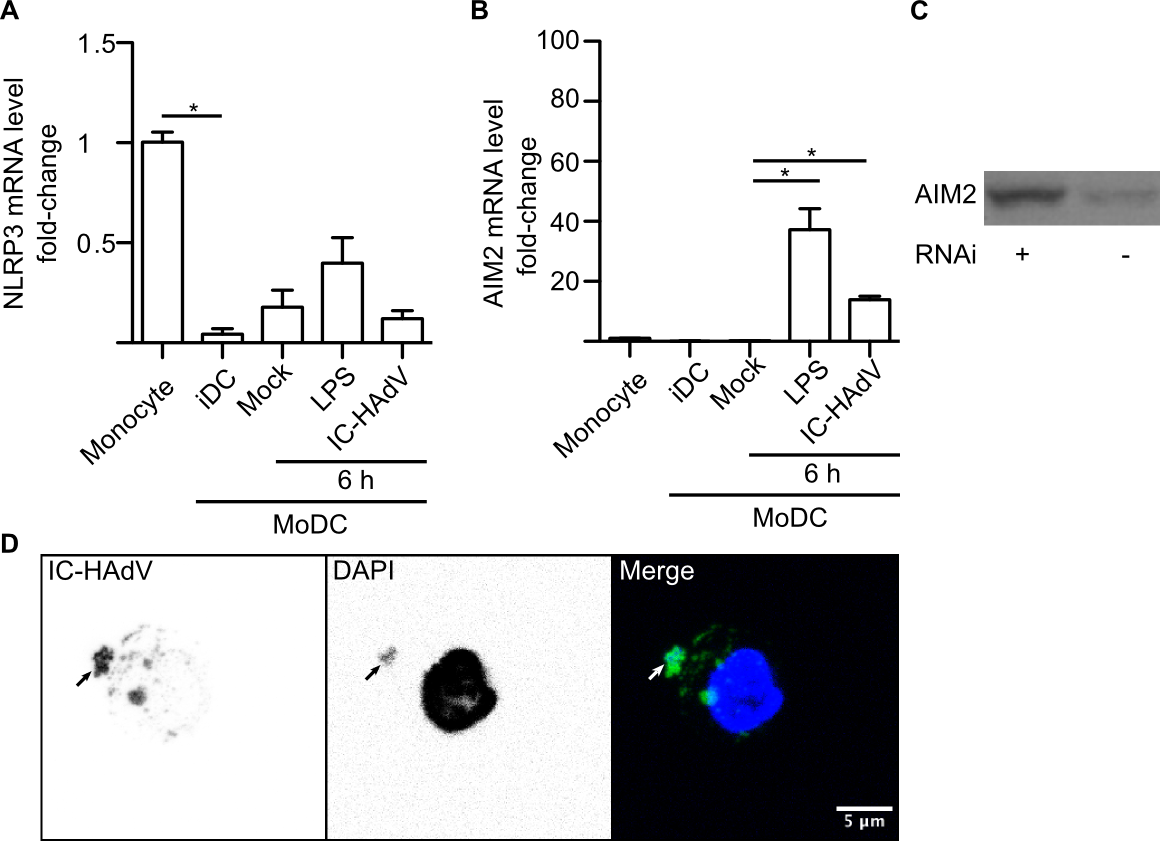

Supplement: S4 Fig — RT-qPCR analysis of A) AIM2 B) and NLRP3 mRNA levels in monocytes and MoDCs and after challenge with LPS or IC-HAdV in MoDC. These assays were performed in triplicate using 3 donors with similar results. C) Immunoblotting demonstrating lentivirus-mediated shRNA knockdown of AIM2 in MoDC. D) Viral DNA is readily detected in the cells and remains associated with viral capsid in IC-HAdV-challenged MoDC. MoDC were exposed to IC-HAdV-488 for 3 h and prepared for fluorescence microscopy with DAPI as counterstaining. (TIF) [file ppat.1005871.s005.tif]

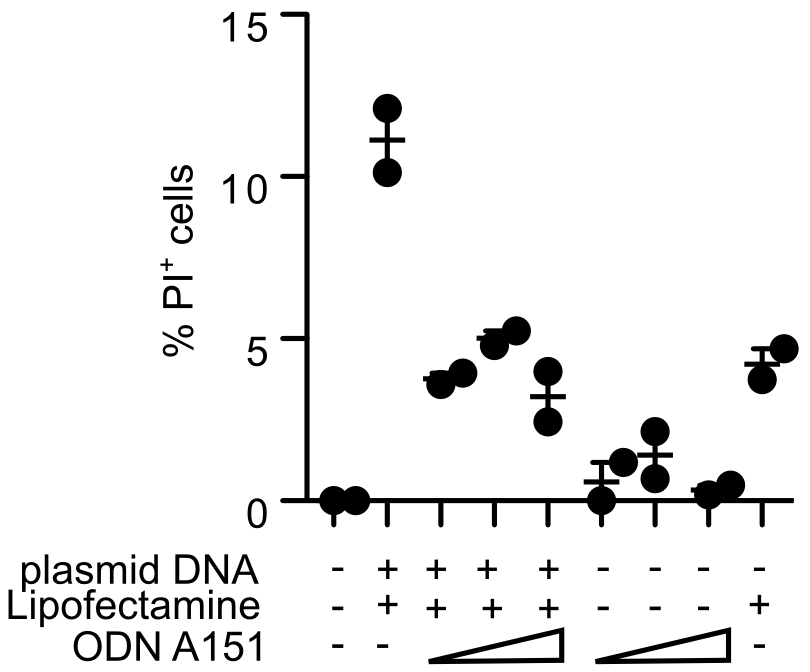

Supplement: S5 Fig — MoDCs were pre-incubated with 10, 50 or 100 μM ODN A151 for 2 h and transfected with plasmid DNA complexed by Lipofectamine LTX and cell membrane integrity was assessed by PI/flow cytometry (n = 2). (TIF) [file ppat.1005871.s006.tif]
